# Supplementary material for: Near real-time data on the human neutralizing antibody landscape to influenza virus to inform vaccine-strain selection in September 2025
Source: bioRxiv. 2025 Sep 6:2025.09.06.674661. Preprint. [Version 1] doi: 10.1101/2025.09.06.674661 (PMC12425000; doi:10.1101/2025.09.06.674661)
Supplement: Supplementary file 1 [file NIHPP2025.09.06.674661V1-supplement-1.pdf]

bioRxiv preprint doi: https://doi.org/10.1101/2025.09.06.674661; this version posted September 6, 2025. The copyright holder for this preprint (which was not certified by peer review) is the author/funder, who has granted bioRxiv a license to display the preprint in perpetuity. It is made available under aCC-BY 4.0 International license.

Supplementary Material

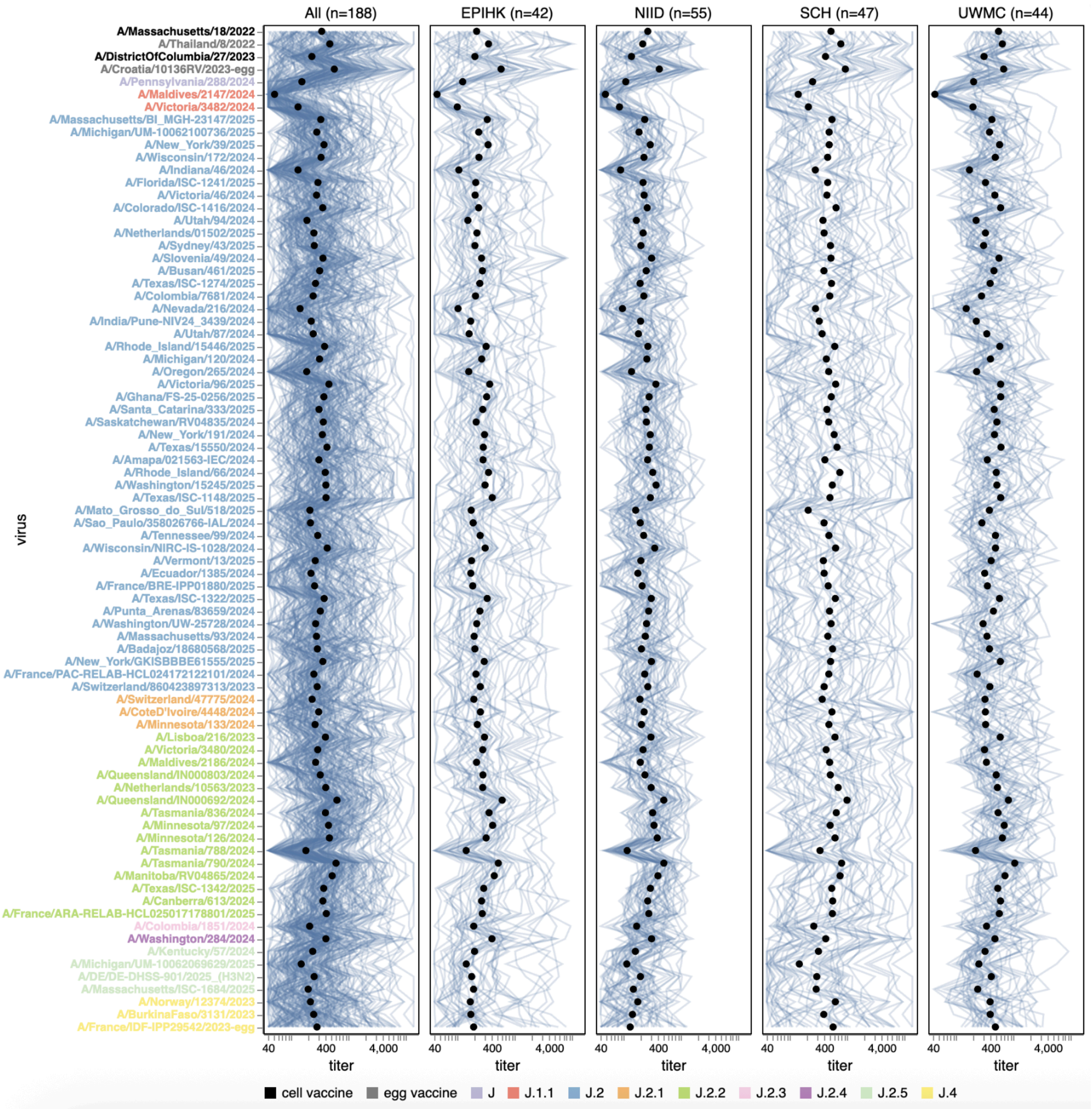

Supplementary Figure S1. Titers of individual human sera to recent H3N2 strains.

Each blue line shows the titers of a serum against the 76 H3N2 strains that capture current circulating diversity, as well as the last two sets of egg- and cell-produced vaccine strains. Black points show the median titer across all sera. Strain labels are colored by whether a strain is a vaccine strain or its subclade per the legend at bottom. The lower limit of detection for titers in our neutralization assays was 40. See https://jbloomlab.github.io/flu-seqneut-2025/human_sera_titers_H3N2_recent_individual_sera.html for an interactive version of this plot that allows mousing over points and lines for details about individual sera or viruses, and subsetting on sera from specific age ranges.

bioRxiv preprint doi: https://doi.org/10.1101/2025.09.06.674661; this version posted September 6, 2025. The copyright holder for this preprint (which was not certified by peer review) is the author/funder, who has granted bioRxiv a license to display the preprint in perpetuity. It is made available under aCC-BY 4.0 International license.

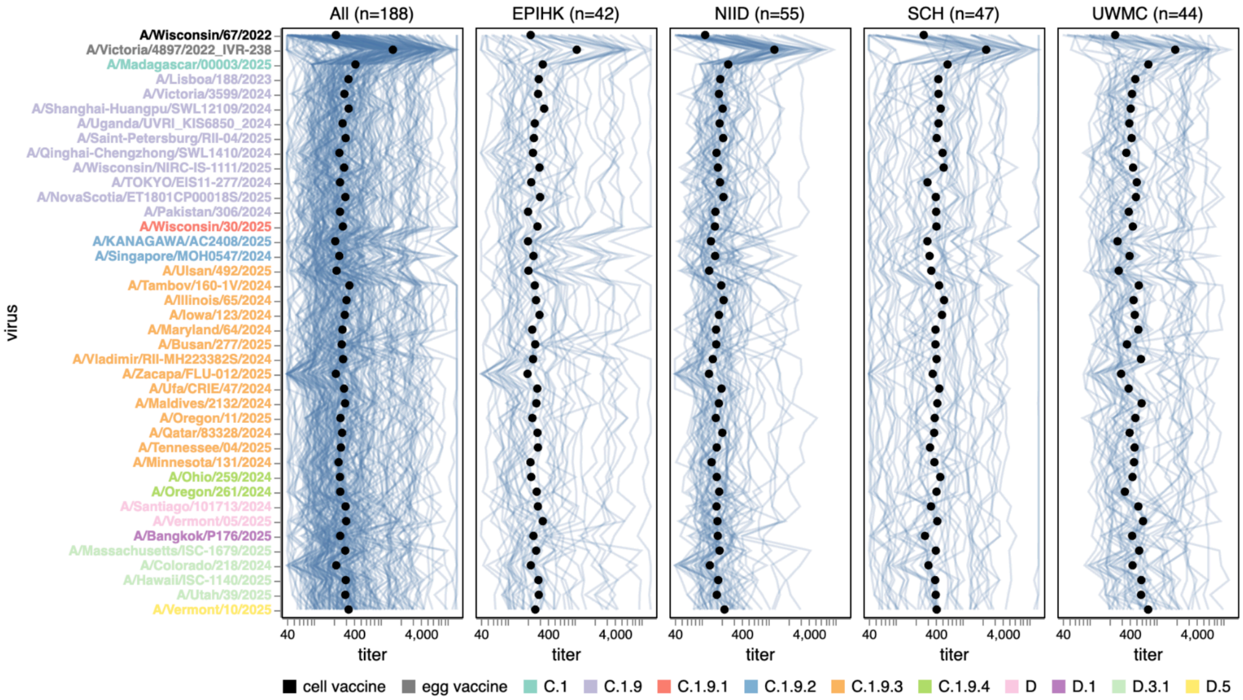

Supplementary Figure S2. Titers of individual human sera to recent H1N1 strains.

Each blue line shows the titers of a different serum against the 38 H1N1 strains that capture current circulating diversity, as well as the last set of cell- and egg-produced vaccine strains. The black points show the median titer across all sera. The leftmost plot shows the titers against all sera, and the remaining plots show titers for sera from each different cohort. Strain labels are colored by whether a strain is a cell- or egg-produced vaccine strain or its subclade per the legend at bottom. The lower limit of detection for titers in our neutralization assays was 40. See https://jbloomlab.github.io/flu-seqneut-2025/human_sera_titers_H1N1_recent_individual_sera.html for an interactive version of this plot that allows mousing over points and lines for details about individual sera or viruses, and subsetting on sera from specific age ranges.

bioRxiv preprint doi: https://doi.org/10.1101/2025.09.06.674661; this version posted September 6, 2025. The copyright holder for this preprint (which was not certified by peer review) is the author/funder, who has granted bioRxiv a license to display the preprint in perpetuity. It is made available under aCC-BY 4.0 International license.

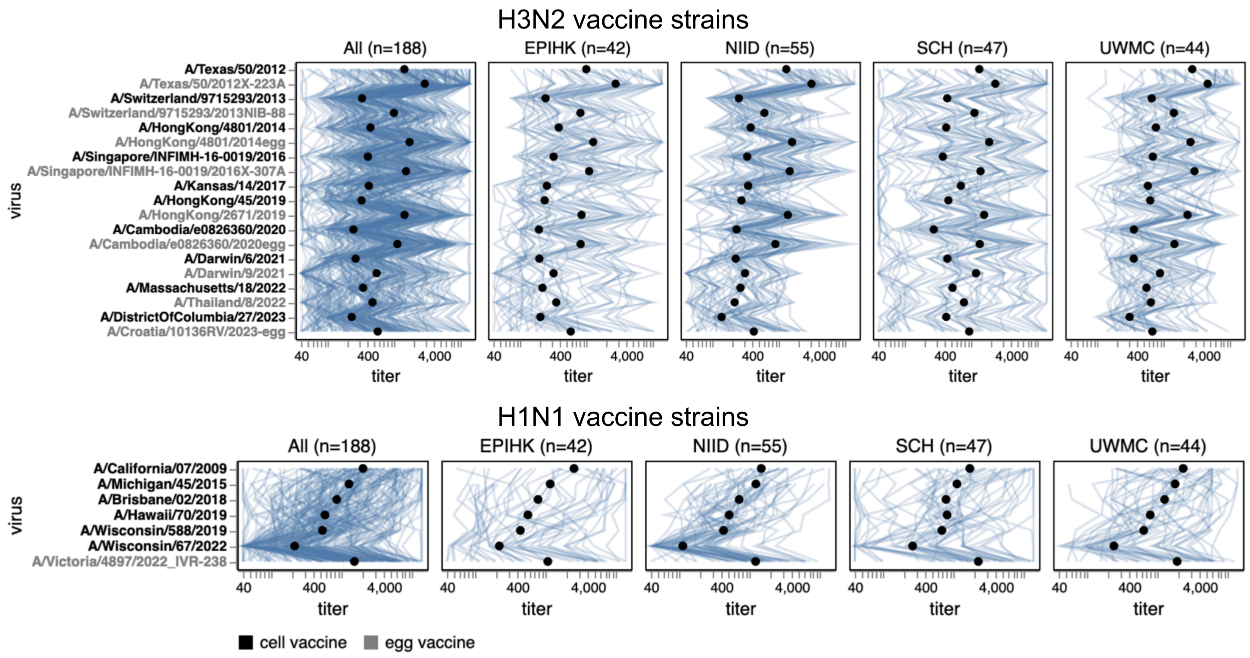

Supplementary Figure S3. Titers of individual human sera to past vaccine strains.

Each blue line shows the titers of a different serum against the viral strains, and the black points show the median titer across all sera. The top panel shows past H3N2 vaccine strains, and the bottom panel shows past H1N1 vaccine strains. The leftmost plot shows the titers against all sera, and the remaining plots show titers for sera from each different cohort. Strain labels are colored by whether a strain is a cell- or egg-produced vaccine strain per the legend at bottom. The lower limit of detection for titers in our neutralization assays was 40. See https://jbloomlab.github.io/flu-seqneut-2025/human_sera_titers_H3N2_vaccine_individual_sera.html and https://jbloomlab.github.io/flu-seqneut-2025/human_sera_titers_H1N1_vaccine_individual_sera.html for an interactive version of this plot that allows mousing over points and lines for details about individual sera or viruses, and subsetting on sera from specific age ranges.

bioRxiv preprint doi: https://doi.org/10.1101/2025.09.06.674661; this version posted September 6, 2025. The copyright holder for this preprint (which was not certified by peer review) is the author/funder, who has granted bioRxiv a license to display the preprint in perpetuity. It is made available under aCC-BY 4.0 International license.

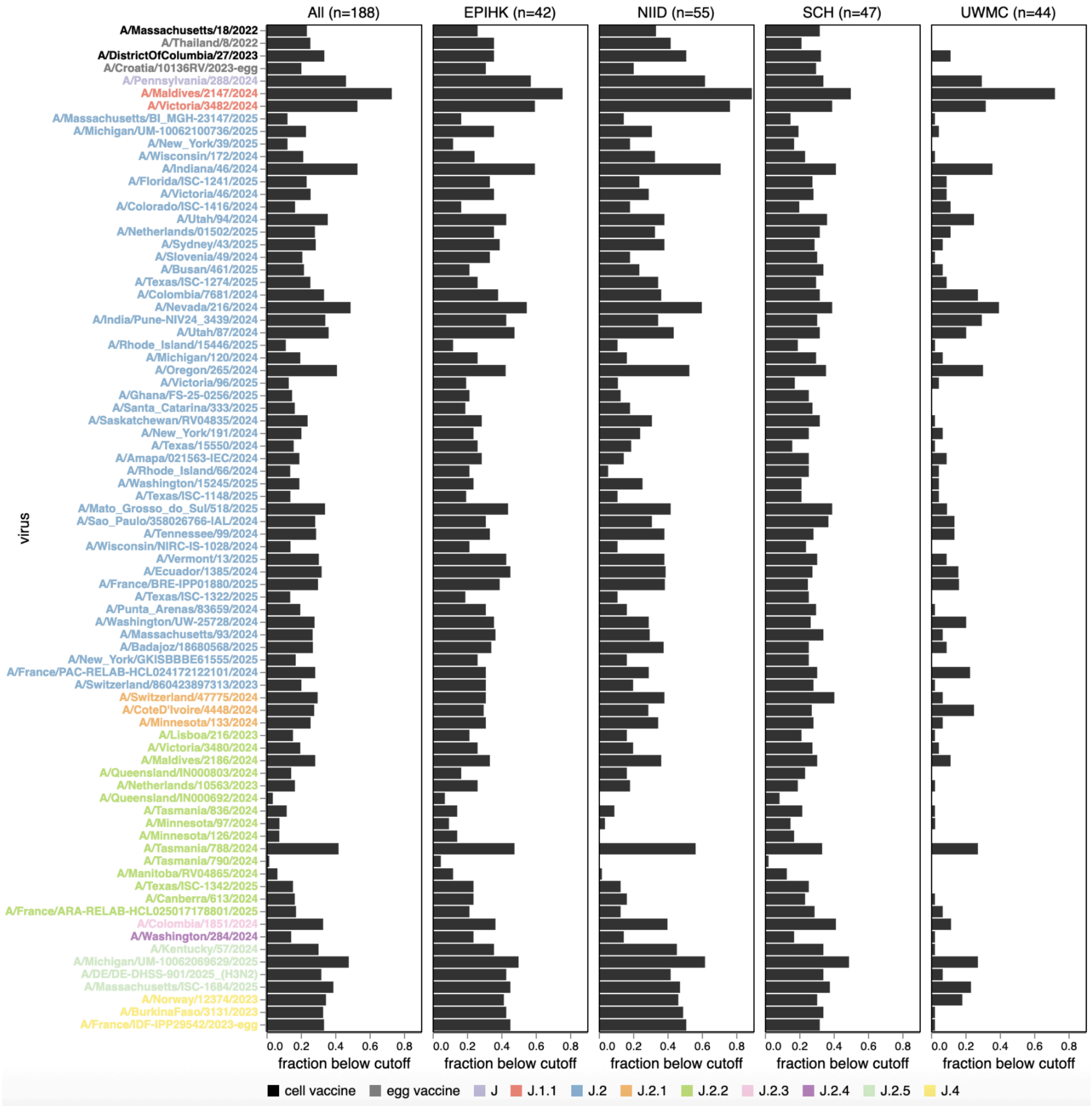

Supplementary Figure S4. Fraction of sera with low titers to each recent H3N2 strain.

Each bar indicates the fraction of sera with a neutralization titer below 140 for each of the 76 H3N2 strains that capture current circulating diversity, as well as the last two sets of egg- and cell-produced vaccine strains. The leftmost panel shows the fractions for all sera, and the other panels show fractions for each subset of sera. Strain labels are colored by whether a strain is a vaccine strain or its subclade per the legend at bottom. See https://jbloomlab.github.io/flu-seqneut-2025/human_sera_titers_H3N2_recent_frac_below_cutoff.html for an interactive version of this figure that enables adjustment of the titer cutoff used to compute the fraction of sera below the cutoff, mousing over points for details, and subsetting on sera from specific age ranges. These values are available as a flat file at

bioRxiv preprint doi: https://doi.org/10.1101/2025.09.06.674661; this version posted September 6, 2025. The copyright holder for this preprint (which was not certified by peer review) is the author/funder, who has granted bioRxiv a license to display the preprint in perpetuity. It is made available under aCC-BY 4.0 International license.

https://github.com/jbloomlab/flu-seqneut-2025/blob/main/results/aggregated_analyses/human_sera_titers_summarized.csv for downstream analysis.

bioRxiv preprint doi: https://doi.org/10.1101/2025.09.06.674661; this version posted September 6, 2025. The copyright holder for this preprint (which was not certified by peer review) is the author/funder, who has granted bioRxiv a license to display the preprint in perpetuity. It is made available under aCC-BY 4.0 International license.

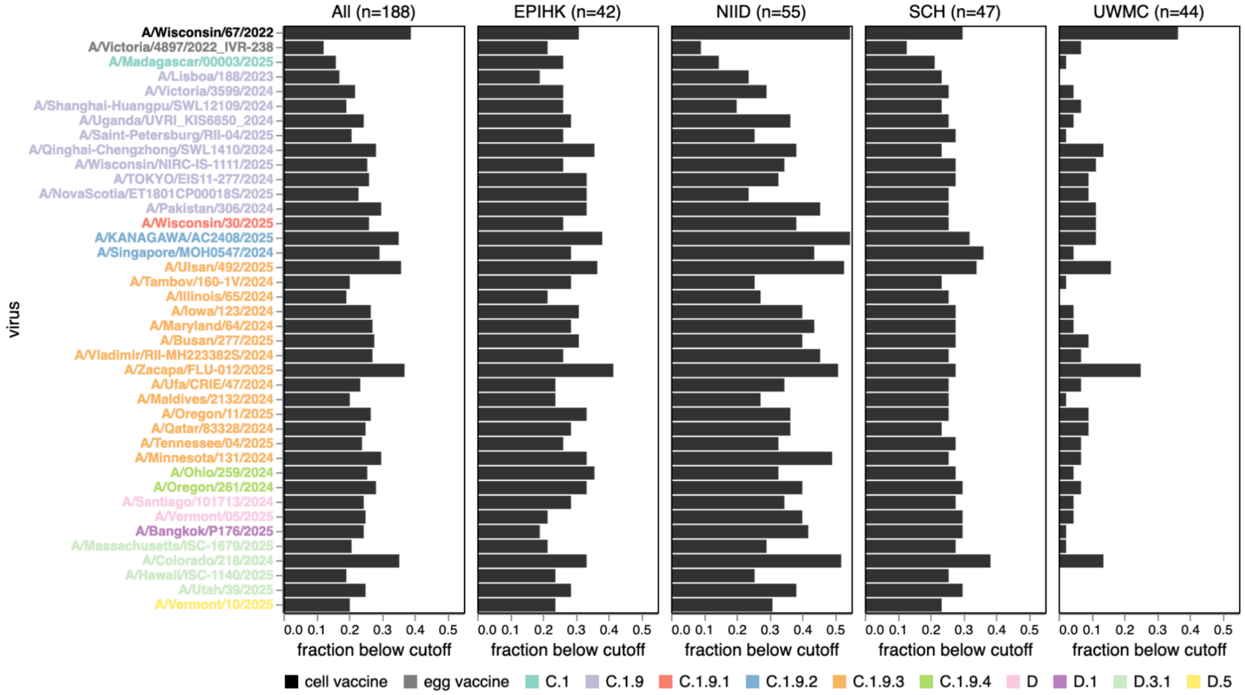

Supplementary Figure S5. Fraction of sera with low titers to each recent H1N1 strain. Each bar indicates the fraction of sera with a neutralization titer below 140 for each of the 38 H1N1 strains that capture current circulating diversity, as well as the last set of egg- and cell-produced vaccine strains. The leftmost panel shows the fractions for all sera, and the other panels show fractions for each subset of sera. Strain labels are colored by whether a strain is a vaccine strain or its subclade per the legend at bottom. See https://jbloomlab.github.io/flu-seqneut-2025/human_sera_titers_H1N1_recent_frac_below_cutoff.html for an interactive version of this figure that enables adjustment of the titer cutoff used to compute the fraction of sera below the cutoff, mousing over points for details, and subsetting on sera from specific age ranges. These values are available as a flatfile at https://github.com/jbloomlab/flu-seqneut-2025/blob/main/results/aggregated_analyses/human_sera_titers_summarized.csv for downstream analysis.

bioRxiv preprint doi: https://doi.org/10.1101/2025.09.06.674661; this version posted September 6, 2025. The copyright holder for this preprint (which was not certified by peer review) is the author/funder, who has granted bioRxiv a license to display the preprint in perpetuity. It is made available under aCC-BY 4.0 International license.

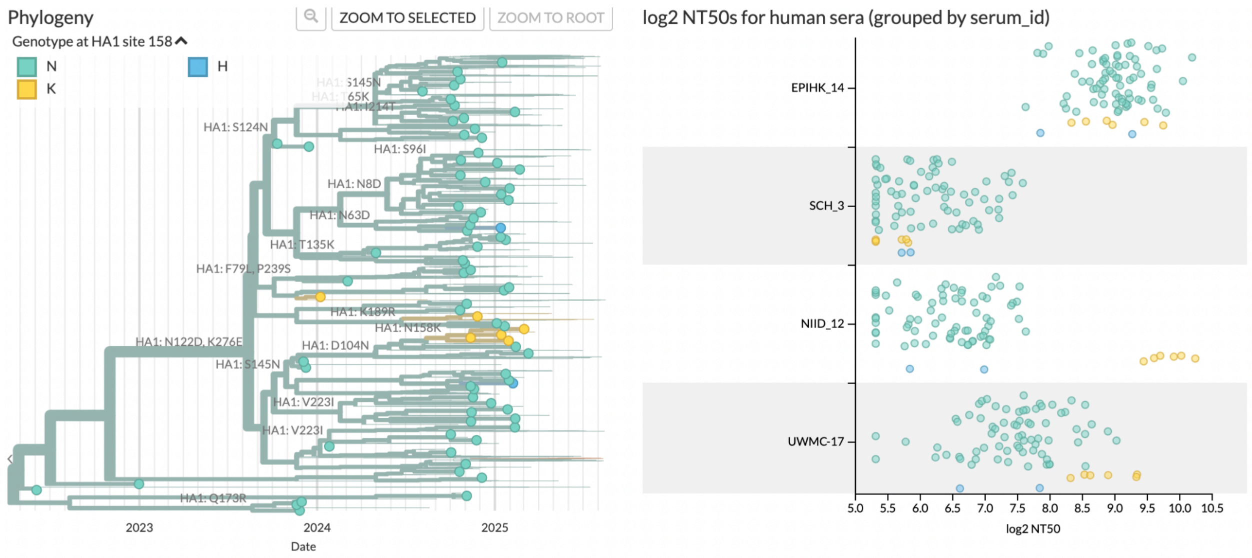

Supplementary Figure S6. Screenshot of Nextstrain interactive visualization showing the H3N2 HA phylogenetic tree (left) and $\log_2$ titer measurements for four serum samples (right) colored by amino-acid identity at HA1 position 158.

This figure demonstrates how the interactive trees can be used to examine how specific mutations affect titers. The phylogeny (identical to that shown in Figure 2 and Figure 8) is filtered to show circles at tip labels only viruses with neutralization titers. The measurements panel shows the variability of titers between and within serum samples with respect to the amino-acid identity at site 158. For example, samples NIID_12 and UWMC-17 have high titers against all 158K viruses, while sample SCH_3 has low titers against 158K and 158H viruses. See https://nextstrain.org/groups/blab/kikawa-seqneut-2025-VCM/h3n2?branchLabel=aa&c=gt-HA1_158&d=tree,measurements&f_kikawa=present_1&focus=selected&gmax=1052&gmin=72&mf_serum_id=EPIHK_14&mf_serum_id=SCH_3&mf_serum_id=NIID_12&mf_serum_id=UWMC-17&p=grid&tl=none for an interactive view of the tree shown here; note that this interactive tree allows similar visualizations for arbitrary mutations or sera.

bioRxiv preprint doi: https://doi.org/10.1101/2025.09.06.674661; this version posted September 6, 2025. The copyright holder for this preprint (which was not certified by peer review) is the author/funder, who has granted bioRxiv a license to display the preprint in perpetuity. It is made available under aCC-BY 4.0 International license.

Supplementary File 1. CSV with details about all sera assayed in the experiments.

This CSV gives details about all sera assayed in the experiments reported here. See https://github.com/jbloomlab/flu-seqneut-2025/blob/main/results/aggregated_analyses/human_sera_metadata.csv for a copy of this CSV.

Supplementary File 2. CSV with details of all strains and barcodes in the library.

This CSV gives the barcode, strain name, nucleotide and protein HA ectodomain sequence, and other details about all viral strains in the library. See https://github.com/jbloomlab/flu-seqneut-2025/blob/main/data/viral_libraries/flu-seqneut-2025-barcode-to-strain_actual.csv for a copy of this CSV.

Supplementary File 3. CSV with all titers measured in the experiments reported here.

This CSV gives the neutralization titers measured in the experiments reported here, averaged across barcodes and replicates for each strain. See https://github.com/jbloomlab/flu-seqneut-2025/blob/main/results/aggregated_analyses/human_sera_titers.csv for a copy of this CSV.
